# Acute phase protein mRNA expressions and enhancement of antioxidant defense system in Black-meated Silkie Fowls supplemented with clove (*Eugenia caryophyllus)* extracts under the influence of chronic heat stress

**DOI:** 10.1186/s40781-016-0122-4

**Published:** 2016-11-30

**Authors:** Alhassan Usman Bello, Jelilat Aderonke Sulaiman, Madagu Samaila Aliyu

**Affiliations:** 1 Institute of Tropical Agriculture, Universiti Putra Malaysia, 43400 Serdang, Malaysia; 2 National Animal Production Research Institute (NAPRI), Shika, Nigeria; 3 University Farm Research, Yobe State University, 1144 Damaturu, Nigeria

**Keywords:** Antioxidant, Acute phase protein, Black-meated fowls, Clove, Immunity

## Abstract

**Background:**

The current study investigates the anti-stress effects of clove (*Eugenia caryophyllus*) extracts (0, 200, 400, and 600 mg/kg) on serum antioxidant biomarkers, immune response, immunological organ growth index, and expression levels of acute phase proteins (APPs); ovotransferrin (OVT), ceruloplasmin (CP), ceruloplasmin (AGP), C-reactive protein (CRP), and serum amyloid-A (SAA) mRNA in the immunological organs of 63-d-old male black-meated Silkie fowls subjected to 21 d chronic heat stress at 35 ± 2 °C.

**Results:**

The results demonstrated that clove extract supplementation in the diet of Silkie fowls subjected to elevated temperature (ET) improve growth performance, immune responses, and suppressed the activities of glutathion peroxidase (GSH-Px), superoxide dismutase (SOD), catalase (CAT), and thioredoxin reductase (TXNRD); reduced serum malonaldehyde (MDA) and glutathione (GSH) concentrations when compared with fowls raised under thermoneutral condition (TC). Upon chronic heat stress and supplementation of clove extracts, the Silkie fowls showed a linear increase in GSH-Px, SOD, CAT, and TXNRD activities (*P* = 0.01) compared with fowls fed diets without clove extract. ET decreased (*P* < 0.05) the growth index of the liver, spleen, bursa of Fabricius and thymus. However, the growth index of the liver, spleen, bursa of Fabricius and thymus increased significantly (*P* < 0.05) which corresponded to an increase in clove supplemented levels. The expression of *OVT, CP, AGP, CRP,* and *SAA* mRNA in the liver, spleen, bursa of Fabricius and thymus were elevated (*P* < 0.01) by ET compared with those maintained at TC. Nevertheless, clove mitigates heat stress-induced overexpression of *OVT, CP, AGP, CRP* and *SAA* mRNA in the immune organs of fowls fed 400 mg clove/kg compared to other groups.

**Conclusions:**

The results showed that clove extracts supplementation decreased oxidative stress in the heat-stressed black-meated fowls by alleviating negative effects of heat stress via improvement in growth performance, antioxidant defense mechanisms, immunity, and regulate the expression of acute phase genes in the liver and immunological organs.

**Electronic supplementary material:**

The online version of this article (doi:10.1186/s40781-016-0122-4) contains supplementary material, which is available to authorized users.

## Background

Stress is simply defined as a biological response of animal species to environmental challenges after their homeostasis threatened [1, 2]. Heat stress deteriorates the productivity and physiological traits in birds [3–6] such as weakening the immunity [7] and increase in mortality rate [8]. Medicinal plants play a vital role in alleviating the effect of heat stress in birds. Clove (*Eugenia caryophyllus*) is reported to contain an essential oil extract characterized by numerous properties [9–13].

Clove extract is colorless to pale yellow oil removed from dried buds characterized with resilient phenolic scent and powerful acrid taste [13]. Clove has been reported to possess strong antioxidant with ability to scavenge free lipid and oxygen radicals [10, 12], contains aphrodisiac activity [11, 12], anti-platelet inhibitory [12, 14], anti-mutagenic, anti-inflammatory, anti-thrombotic [14], anesthetic [15], anti-bacterial [16], and anti-hyperlipidemic [17].

It has been showed that certain polyphenolic supplements can regulate acute phase response (APR) mRNA expression [18]. APR is an innate and homeostatic response to tissue injury, stress and infection associated with physiology, immune, and endocrine processes [19]. APR is defined by a distinct change in the plasma concentrations of the APPs. The variation in these proteins can be either minor or major, positive or negative [20, 21]. The classical avian APPs are AGP, OVT, SAA, CP, and CRP. Primary actions of positive APPs are on hemoglobin, free radicals, immunoglobulin synthesis, and removal of aggregate cells. Positive APPs mRNA synthesis is correlated with a substantial decrease in synthesis of common serum proteins (i.e., pre-albumin and albumin, the negative APPs) [22]. The initial stage of inflammation (the AP response) is defined by local and systemic stimulation of cells and secretion of soluble mediators of inflammation which aggregate the metabolic response of the whole body [22]. The important indicator of this reaction is the induction of APPs genes expression in the liver, and other organs ensued from the interactions of specific transcription factors with hormone reactive elements in the promoter and enhancer of the mark APPs genes [23]. Similarly, Fulop [24] indicated that during the acute phase response, increase synthesis of APPs in the liver is measured by an increased transcription of APPs mRNA and elevation of their serum concentrations. In a recent study by Li et al. [25] using a breast muscle and a liver tissue cDNA library from Silkie fowl by measuring the global gene response to chronic heat stress, found a new heat-reactive genes such as mitogen-activated protein kinase that play a significant role in heat regulation.

Silkie fowls are type of chickens with black skin, bones and grayish-black meat, dark blue wattle, comb, beak, and toes [26]. This color phenomenon is as a result of hyperpigmentation of melanin content of these tissues and organs. Studies have indicated that melanin has an antioxidant effect [27, 28]. Melanin extracts has reduced and chelating power on Ferrous (Fe^2+^). Antioxidant effects of melanin extracts has a complex mechanism and functions associated to stress in animals [29]. Typical Silkies are fairly small fowls, with the males weighing 1.1 to 1.8 kg while the females weigh 0.9 to 1.11 kg [30]. Several scientists have studied characteristics of Silkie fowls [31–34]. However, little or no data uncovered so far in connection with heat stress.

When bird is subjected to heat stress, it often show a weaken immune system which makes them prone to a variety of infections that may results in poor productivity. However, the mechanisms underlying heat stress induced APR mRNA expression in the immune organs of black Silkie fowls is unobtainable. Studies have revealed that APPs protect DNA from oxidative damage [18, 35, 36], where clove may serve as possible DNA protector. The mechanism by which the elevated temperatures suppress immunity is not completely understood. No previous attempt made to use clove to neutralize the phenomenon of heat stress in Silkie fowls. Therefore, it is practical to hypothesize that clove may decrease cellular malonaldehyde and heat stress-induced reactive oxygen species in the black Silkie fowls. Therefore, current study was carried out to investigate the anti-stress effects of clove extracts on immune organ growth index upon oxidative damage and antioxidant biomarkers concentrations as well as a chaperone inducer that enhances the regulation of APPs mRNA expression in these immune organs of black-meated Silkie fowls subjected to chronic heat stress.

## Methods

### Bird husbandry, diets, and experimental design

Total of one hundred and seventy-five (175) male black-meated Japanese Silkie fowls, Ukokkei (35 d old) with well-documented history and free from infectious diseases, were obtained from Local Commercial Fowl breeder Company and relocated to the research facility of the National Animal Production Research Institute, Shika. Clove (*Eugenia caryophyllus*) extract (≥98% purity contains) was acquired from Agricultural Research Institute, Zaria. All fowls were allowed access to basal diet and water *ad libitum*. The basal diet was formulated to meet NRC [55] requirement (Table 1). After seven days adaptation period, each black-meated fowl was separately weighed (the average weight was 591 g). All birds were then distributed into **five** groups at random; each group of black-meated fowls was further subdivided into **seven** replicates (**five** fowls/replicate) and raised in seven cages with a dimension of 100 × 115 cm. Group one, 35 black-meated fowls were fed the basal diet and kept at 24 ± 2 °C for 24 h/d (thermoneutral condition, **TC**). Then, the remaining **four** groups (140 black-meated fowls) were kept in temperature-regulated rooms at 35 ± 2 °C for 8 h/d (elevated temperature, **ET**; 0900–1700 h) followed by maintaining at 24 ± 2 °C for the remaining 16 h/d. The treatment sustained for 21 d (from d 42 to 63). During this period, the fowls of **TC** group assigned the formulated basal diet only, while the fowls of **ET** groups were allocated 1 of 4 diets: either the basal diet or basal diet plus 200, 400, or 600 mg clove extract per kg of diet. Upon 63 d, blood samples, liver, and immunological organs were sampled from two bird per replicated cage for further laboratory analysis (70 birds, 14 birds per each of the five groups).

**Table 1:** Experimental basal diet compositions^c^ (air-dry basis)

Item	Quantity (%)
Ingredient	
Corn	60.95
Soybean meal	28.34
Wheat offal	3.28
Oyster shells	1.22
Palm oil	3.25
Dicalcium phosphate	1.66
Salt	0.35
Antioxidant	0.21
Vitamin and mineral premix^a^	0.54
Chloride choline	0.12
D, L-Methionine	0.08
Total	100
Nutrient concentrations^b^	
ME, MJ/kg	12.66
CP, g/kg	18.58
Phosphorus, g/kg	0.48
Calcium, g/kg	1.04
Methionine, g/kg	0.37
Lysine, g/kg	0.82

^a^The premix supplied per kilogram of diet: vitamin A, 12,550 IU; vitamin D_3_, 3,010 IU; vitamin E, 26 IU; vitamin B_1_, 3 mg; vitamin B_2_, 6.7 mg; Vitamin B_12_, 0.22 mg; Vitamin K_3_, 3.24 mg; Biotin, 0.09 mg; folic acid, 1.4 mg; d-pantothenic acid, 12.6 mg; nicotinic acid, 44 mg; copper, 8 mg; iron, 80 mg; zinc, 42 mg; Manganese, 60 mg; Selenium, 0.17 mg; iodine, 0.35 mg

^b^Nutrient concentrations: ME was a calculated value, whereas other nutrient concentrations were determined values

^c^NRC requirement [55]

### Growth performance

The BW and feed intake were measured on daily basis by cage. Feed conversion was calculated as the feed to gain ratio. The gain, FI, and F/G were corrected by mortality.

### Measurement of liver and immunological organ growth index

The fowls were weighed and slaughtered. The liver, spleen, bursa, and thymus were severed and weighed. The immune organ index (mg/g) was calculated as liver or immune organ fresh weight (mg)/fowl weight (g) before slaughter.

### Determination of antioxidant biomarkers

The activities of catalase (**CAT**), glutathione peroxidase (**GSH-PX**), thioredoxin reductase (**TXNRD**), superoxide dismutase (**SOD**), and concentrations of glutathione (**GSH**) and malonaldehyde (**MDA**) in the serum were measured according to the instructions supplied with the commercial assay kits (Cell Biolabs, Inc., San Diego, USA). Measurements were taken using an automated fluorescence spectrometer (BioTek Instruments, Inc., USA). The TBA procedure was used to quantify the MDA concentration at a wavelength of 532 nm (Yang et al., 2010). The GSH concentration was measured at wavelength 405 nm colorimetrically detecting the glutathione reaction with 5,5′-dithio-bis-2-nitro-benzoic acid [37]. The action of SOD was measured by the nitrite coloration method and the absorbance at 450 nm. The activity of CAT was measured at wavelength 405 nm via ammonium molybdate technique. The activities of GSHPx was determined at 412 nm by measuring the rate of oxidation of the reduced GSH to oxidized glutathione [38].

### Real-Time PCR (Quantitative)

Total RNA from the liver, bursa of Fabricius, spleen, and thymus tissues was isolated using TRI Reagent® (Sigma-Aldrich, Co. LLC.), and the cDNA was synthesized from 1 μg of RNA with an iScript cDNA Synthesis Kit (BioRad, Lagos, NG) in accordance with manufacturer’s directives. Primer pairs of sense and antisense were designed on the basis of cloned complete sequences of *β-actin* (GenBank accession no. L08165.1), *APG* (no. AY584568.1), *OVT* (no. NM_205304), *CP* (no. XM_015291853.1), *CRP* (no. DQ374639.1), and *SAA* (no. GU929209.1) from *Gallus gallus domesticus* for the real-time quantitative PCR (Table 2). The β-actin gene was chosen as the reference gene. All the primers were synthesized and purified by Inqaba Biotechnical Industry (West Africa Ltd., NG). The PCR amplification reaction had 1 μL of cDNA template, 12.5 μL of DreamTaq Green 2× PCR Master-Mix (Thermo-Fisher Scientific Inc.), 1 μL of each of the upstream and downstream primers, and 10.5 μL of sterilized deionized water making a total reaction volume of 25 μL. The amplification factors of the thermocycler (Biometra GmbH, Göttingen, Germany) were a preheat for 5 min at 94 °C, then 40 cycles for 30 s at 94 °C, follow by 40 s at 59 °C, then 1 min at 72 °C, and the final extension at 72 °C for 10 min. Subsequently, 4 μL of PCR product was pipetted onto a 0.9% agarose gel and analyzed via gel electrophoresis. The quantitative real-time PCR reactions were done by taken 10 μL of 2× SYBR Premix EX Taq Super Mix (Sigma-Aldrich, Co. LLC.), 0.4 μL of 50× ROX Reference Dye, 1 μL of cDNA template, 7.8 μL of sterilized water, and 0.8 μL each of the forward and reverse primers (10 μ*M*) making total reaction volume of 20 μL. The PCR were conducted on an ABI Prism 7900HT Sequence Detection System (ThermoFisher Scientific, CA, USA). The thermal cycler factors used were 30 s at 95 °C; 40 cycles for 5 s at 95 °C, then 31 s at 59 °C, and then extra detachment cycle of 15 s at 95 °C, followed by 60 s at 60 °C, then 15 s at 95 °C, and 15 s at 60 °C. However, the target gene expression was standardized to that of the selected reference gene, and the relative gene expression was measured using the 2 − ^ΔΔCT^ method of Livak and Schmittgen [39]. The threshold cycle values were deduced from the cycle number at which the specific gene was amplified beyond the selected threshold.

**Table 2:** Oligonucleotide primers Sequences for acute phase proteins

Name	Fragment size (base pairs)	Sense	Antisense
β-actin	1736	5′-AGCCAACAGAGAGAAGATGAC-3′	5′-CATCACCAGAGTCCATCACAA-3′
OVT	2376	5′-ATCACAGACCCAGAGGGGACG-3′	5′-CCCAACATGAAGCTCATCCTC-3′
CP	4134	5′-GAGAGTAAGGGTGGGGGTGGG-3′	5′-TATTTCACATTTTCCACAAGG-3′
AGP	814	5′-TCTGATCTAGACCTGCAGGCTC-3′	5′-ATCCTCGCCATGGGGTTGGTG-3′
CRP	684	5′-ATGGGCACCGCGCCGATGTGG-3′	5′-ACAGAAAGGTGTTTGTGTTCC-3′
SAA	384	5′-ATGAGGCTTTGTATCTGCTTCG-3′	5′-GCGGTATCAAGTTTGTCAGGG-3′

### Blood samples analyses

Blood samples were centrifuged at 2,000 × *g* at 4 °C for 15 min to separate the plasma and the supernatant were stored at −30 °C until the plasma constituent was measured. The blood leucocytes were analyzed using a Multi-species Hematology System (S/N HV03234, Hemavet 950, Drew Scientific Inc., Oxford). The plasma IgA, IgM, and IgG were measured using chicken IgA, IgM, and IgG ELISA Kits, respectively (Abcam, Cambridge, MA, USA) as previously described by [40].

### Statistical analysis

All data were statistically analyzed using a completely randomized design (CRD) with the cage serves as experimental unit using GLM method of SAS (Version 9.3, SAS Institute Inc., Cary, NC). A single degree of freedom contrast was applied to determine the effect of **ET** versus **TC** for the fowls placed on a basal diet without clove supplement. Orthogonal polynomial contrasts were applied to measure the linear and quadratic responses of the black-meated fowls supplemented clove levels in the **ET** environment. Probability 0.05 level was considered statistical significance.

## Results

### Growth performance

The effects of clove supplementation on broiler performance are shown in Table 3. It significantly (P < 0.01) increased BW, ADG, ADFI and lowered F/G (P < 0.05) in birds supplemented diet containing clove under elevated temperature compared with those maintained under thermoneutral condition during 42–63 period. No clove*heat interaction for BW, ADG, ADFI, and F/G was noted throughout the 21-d trial.

**Table 3:** Effects of clove supplementation on growth performance of the black-meated fowls^1^ under elevated temperature

Parameter	Clove inclusion level (mg/kg)				PSEM^2^	P-value	Temp*Clove
	0	200	400	600			
Initial BW (g)	591.1	590.9	591.2	590.0	0.1		
TC^3^ group							
d 42-63							
BW (g)	1183.4^a^	1153.7^a^	1252.7^b^	1297.7^b^	18	0.002	0.2403
ADG (g)	28.2^a^	26.8^ab^	31.5^b^	33.7^b^	0.2	0.003	0.1372
ADFI (g)	36.2^a^	34.4^a^	37.5^b^	43.0^b^	0.5	0.013	0.0670
F/G	1.28 ^a^	1.28^a^	1.19^c^	1.13^c^	0.03	0.0042	0.4219
ET^4^ group							
d 42-63							
BW (g)	1107.6^a^	1244.0^a^	1353.4^b^	1388.0^b^	23	<0.0001	0.1063
ADG (g)	24.6^a^	31.1^ab^	36.3^b^	38.0^b^	0.3	0.011	0.1456
ADFI (g)	32.9^a^	37.4^a^	41.5^b^	43.0^b^	0.4	0.001	0.0011
F/G	1.34^b^	1.20^a^	1.14^c^	1.13^c^	0.012	0.0033	0.0501

^a-c^ Means with different superscript in a column are significantly different at *p* < 0.01

^1^Data are means of seven replicates. ^2^PSEM = Pooled standard error of the mean. Temp = temperature

^3^ TC: Thermoneutral temperature treatment (24 ± 2 °C); ^4^ ET: Elevated temperature treatment (35 ± 2 °C)

### Liver and immunological organs growth index

The effects of chronic heat stress and clove supplementation on the growth index of the liver, spleen, bursa of Fabricius, and thymus are presented in Table 4. The index of the liver of ET groups was significantly (*P* < 0.004) increased by an increase in clove levels. The fowls supplemented with 400 mg/kg clove level exhibited similar liver index compared those kept at TC. The bursa of Fabricius growth index was not significantly affected by the chronic heat stress. However, the index of the spleen and thymus were reduced (*P* < 0.05) in the ET groups provided a diet without grounded clove compared with fowls kept under TC. Though, there was linear increased in the index of spleen and thymus upon ET treatment and supplemented clove extract which correlated with increased levels of supplemented clove (*P* < 0.05). The growth index of the spleen and thymus showed highest values for the Silkie fowls supplemented 400 mg clove in the diet following the ET treatment.

**Table 4:** Effect of elevated temperature and clove supplementation on immunological organs of black-meated fowls from 42 to 63 d^a^

Treatment		Immune organ growth index (mg/g)		
	Liver (g/bird)	Spleen	Bursa of Fabricius	Thymus
TC,^b^ added clove, mg/kg				
0	32.68	1.30	3.99	1.13
ET,^b^ added clove, mg/kg				
0	27.87	1.18	3.53	0.90
200	29.72	1.34	4.32	1.00
400	33.01	1.51	4.68	1.12
600	35.34	1.55	5.66	1.17
SEM	0.68	0.04	0.13	0.03
	*P*-value			
TC vs. ET at 0 mg of clove/kg	0.043	0.016	0.140	0.003
Effects of clove under ET				
Linear	0.004	0.014	0.039	0.025
Quadratic	0.093	0.148	0.341	0.007

^a^Data are means of 2 birds per replicate (5 birds per cage; 7 replicates per group). ^b^TC: Thermoneutral temperature treatment (24 ± 2 °C); ET: Elevated temperature treatment (35 ± 2 °C)

### Serum antioxidant and oxidative biomarkers

The serum TXNRD activity and MDA concentrations were higher (*P* < 0.001) in the ET groups compared with TC groups (Table 5). The black-meated fowls subjected to ET exhibited approximately 25 and 66% more serum TXNRD and MDA respectively than those of TC fowls. A linear increased (*P* < 0.001) in serum TXNRD with decrease (*P* < 0.001) in the MDA level was noted for the ET clove supplemented groups. In contrast, serum GSH-Px, CAT, SOD activities and GSH concentrations were lower (*P* < 0.001) in the fowls challenge to ET compared with fowls kept under TC. There was linear increased (*P* ≤ 0.01) in serum GSH-Px, CAT, SOD activities and GSH concentration (Table 5) in the clove supplemented groups. However, serum GSH-Px (*P* < 0.001), CAT (*P* < 0.001) and SOD (*P* < 0.001) activities demonstrated a significant quadratic response with increase in clove levels. These results indicate that clove supplementation increased (*P* < 0.01) the antioxidant enzymes activities and reduced (*P* < 0.001) the concentration of products of lipid peroxidation in the ET black-meated fowls.

**Table 5:** Effect of elevated temperature and clove supplementation on the antioxidant enzyme activities and oxidative index of black-meated fowlsa

Treatment	Antioxidant and Oxidative index					
	MDA,^c^ nmol/mL	GSH,^c^ nmol/mL	GSH-Px,^c^ 10^2^ units/mL	SOD,^c^ units/mL	CAT,^c^ units/mL	TXNRD units/mL
TC,^b^ added clove, mg/kg						
0	3.59	1.79	23.67	220.61	61.08	40.13
ET,^b^ added clove, mg/kg						
0	5.23	0.72	15.83	173.29	32.12	50.89
200	4.30	1.32	18.49	196.11	57.00	55.04
400	3.61	1.55	23.77	221.97	55.01	57.99
600	3.52	1.86	24.53	231.86	65.00	60.07
SEM	0.101	0.058	2.83	3.09	2.12	1.89
	*P*-value					
TC vs. ET at 0 mg of clove/kg	0.001	<0.001	<0.001	0.001	<0.001	<0.001
Effects of clove under ET						
Linear	<0.001	0.01	0.012	0.014	0.012	<0.001
Quadratic	0.075	0.003	<0.001	0.009	<0.001	0.004

^a^Data are means of 2 birds per replicate (5 birds per cage; 7 replicates per group). ^b^TC: Thermoneutral temperature treatment (24 ± 2 °C); ET: Elevated temperature treatment (35 ± 2 °C). ^c^MDA = malondialdehyde; CAT = catalase; GSH-Px = GSH peroxidase; TXNRD = thioredoxin reductase

### Acute phase protein mRNA Expression Levels

**Fig. 1:**
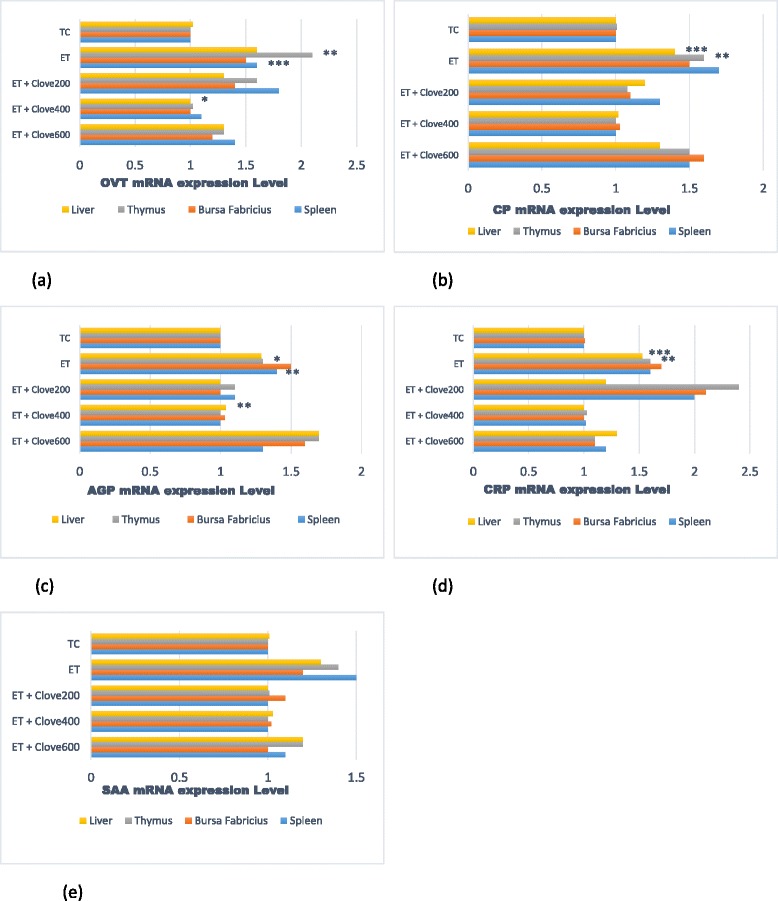
Effects of ET and clove levels on APP; *OVT, CP, AGP, CRP* and *SAA* mRNA expression. Values are Means ± SEM, *n* = 2. The *OVT, CP, AGP, CRP,* and *SAA* mRNA expression of ET (clove, 0 mg/kg) was different from TC, *P < 0.05, **P < 0.01, ***P < 0.001 (ET vs. TC, without clove). **a** Linear contrasts of 0, 200, 400, & 600 mg clove of *OVT* mRNA expression of the liver, bursa of fabricius, thymus, and spleen are expressed as *P* = 0.001, *P* = 0.002, and *P* = 0.008, respectively, and the quadratic contrast of clove as *P* = 0.05, *P* = 0.172, and *P* = 0.0046, respectively. **b** Linear contrasts of 0, 200, 400, and 600 mg clove of *CP* mRNA expression of the same organs are expressed as *P* < 0.002, *P* = 0.004, and *P* < 0.001, and the quadratic contrasts of clove as *P* = 0.001, *P* = 0.471, and *P* < 0.003. **c** Linear contrasts of 0, 200, 400, and 600 mg clove of *AGP* mRNA expression of the same organs are expressed as *P* < 0.002, *P* = 0.012, and *P* < 0.001, and the quadratic contrast of clove as *P* = 0.001, *P* = 0.049, and *P* = 0.001, respectively. **d** Linear contrasts of 0, 200, 400, and 600 mg clove of *CRP* mRNA expression of the same organs are expressed as *P* < 0.001, *P* = 0.031, and *P* < 0.003, and the quadratic contrast of clove as *P* = 0.003, *P* = 0.033, and *P* = 0.001, respectively. **e** Linear contrasts of 0, 200, 400, & 600 mg clove in *SAA* mRNA expression of the same organs are expressed as *P* < 0.004, *P* = 0.054, and *P* < 0.001, and the quadratic contrast of clove as *P* = 0.001, *P* = 0.01, and *P* = 0.029, respectively

The differences in the induced *OVT, CP, AGP, CRP* and *SAA* mRNA expression were distinct among the measured organs of the treated fowls (Fig. 1 a, b, c, d, & e). All organs; liver, spleen, bursa of Fabricius, and thymus exhibited comparable *OVT, CP, AGP, CRP* and *SAA* mRNA induced characteristics under ET. The mRNA expression levels of *OVT, CP, AGP, CRP* and *SAA* of all organs (*P* ≤ 0.01) were greater (*P* < 0.05) in black-meated fowls subjected to ET without the clove supplementation when compared with fowls raised under thermoneutral condition. On the contrary, *OVT, CP, AGP, CRP* and *SAA* mRNA expression in the liver, spleen, and thymus were decreased in the ET-treated fowls fed with dietary clove. The expression of *OVT, CP, AGP, CRP* and *SAA* in the measured organs were lower significantly (*P* < 0.001) in ET-treated fowls supplemented with 400 mg clove compared to other levels. The data acquired from birds supplemented 400 mg clove/kg under ET were very close to those obtained from fowls reared under TC. These mRNA expression levels displayed a significant quadratic response (*P* = 0.003, *P* < 0.001, *P* = 0.012, *P* < 0.001, and *P* = 0.001 respectively) to increasing levels of the dietary clove supplementations. Nevertheless, the bursa Fabricius and liver showed lower (*P* < 0.05) *OVT, CP,* and *CRP* mRNA induction in response to chronic heat stress compared to expression in other organs. However, *OVT, CP,* and *SAA* mRNA were expressed at a higher level in the spleen and thymus (Fig. 1 a, b, & e). The mRNA expression of *AGP* and *CRP* in the spleen was lower in fowls exposed to ET compared to those maintained in TC (*P* < 0.01). An increase in *SAA* mRNA expression in the bursa Fabricius correlated with increased doses of clove supplements; while the mRNA expression in the liver showed an inverse correlation with clove doses. Expression of *CP, AGP, and SAA* mRNA were at their peaked for liver, thymus, and bursa Fabricius of ET-treated black-meated fowls supplemented 600 mg/kg clove in the diet.

### Immunological responses

The immunological responses after heat challenge are presented in Tables 6 and 7. The white blood cells count was significantly greater in 600 mg clove supplemented group compared with other ET groups and TC fowls. The monocyte and eosinophils concentrations were also greater in 600 mg clove supplemented group compared with TC fowls. However, no significant difference was noted among the treatments for other compositions of blood leucocytes of the fowls (Table 6). The dietary clove level of 400 and 600 mg/kg supplemented significantly (*P* < 0.05) elevated the plasma immunoglobulin concentrations (IgA, IgM, and IgG), where a greater level was noted for 600 mg clove as compared with other treated groups (Table 7).

**Table 6:** Effect of clove extract supplementation on blood leucocytes of black-meated fowls (21 d)^a^

Parameter	Treatment ET^b^ (supplemented clove, mg/kg)					
(%)	TC^c^	0	200	400	600	SEM
White blood cells	0.65^b^	0.83^b^	1.35^ab^	2.23^a^	3.15^a^	0.19
Heterophils	24.1	18.6	19.7	21.2	25.4	1.15
Lymphocytes	72.6	71.1	71.9	72.5	73.2	1.17
Heterophil:Lymphocyte	0.31	0.24	0.25	0.30	0.32	0.06
Monocytes	2.34^b^	4.30^ab^	5.12^ab^	5.73^a^	6.18^a^	0.33
Eosinophils	2.29	1.69	3.07	2.97	3.05	0.38

^a^ Data are means of 2 birds per replicate (5 birds per cage; 7 replicates per group). ^a,b^Means with common superscripts within a row not differ significantly (*P* < 0.05)

^b^ ET: Elevated temperature treatment (35 ± 2 °C); ^c^ TC: Thermoneutral temperature treatment (24 ± 2 °C)

**Table 7:** Effect of clove extract supplementation on plasma immunoglobulin levels of black-meated fowls (21 d)^a^

	Treatment^b^ ET (supplemented clove, mg/kg)					
Immunoglobulin						
(μg/mL)	TC^c^	0	200	400	600	SEM
IgA	273.6^c^	280.5^c^	313.2^b^	378.7^a^	387.5^a^	10.02
IgG	522.1^c^	595.0^c^	601.9^b^	642.6^a^	658.3^a^	9.21
IgM	66.59^c^	70.11^c^	79.04^b^	84.23^a^	90.42^a^	2.48

^a^ Data are means of 2 birds per replicate (5 birds per cage; 7 replicates per group). ^a–c^Means with common superscripts within a row not differ significantly (*P* < 0.05)

^b^ ET: Elevated temperature treatment (35 ± 2 °C); ^c^ TC: Thermoneutral temperature treatment (24 ± 2 °C)

## Discussion

### Growth performance

The results of this experiment showed that clove extract had a greater effect on the growth performance under elevated temperature. Highest BW, ADG, ADFI, and lower F/G were recorded for ET group, which was significantly differs (P < 0.01) from TC group. The ET by clove interactions values were significantly higher in ADFI (P < 0.001) and F/G (P < 0.05), where no significant interactions was noted for control. Clove as a digestive stimulant has been previously reported to provide beneficial effect [57]. Its stimulating effects on performance [58, 59] and digestive enzymes [60] of birds have been reported. The clove stimulants which are mostly antioxidants could be ascribed to its phytochemical contents providing protective effect [61] or due to their trace element contents which are prerequisite for the antioxidant enzyme activities [63] thereby promoting the performance. Though combination of clove extract and temperature supported higher growth than under thermoneutral condition.

### Antioxidant defense mechanisms

The term antioxidant is any substance that, when exists at high concentration, compared with those of the oxidizable substrate, significantly impedes or delays oxidation of the substrate [41]. When pro-oxidants increase and antioxidants level decrease, resulted in a condition of oxidative stress that may lead to excessive molecular and tissue damage [41]. Clove is considered a vital monomeric bioactive substance that demonstrates high antioxidant properties [42] and acts as an anti-stress to improve tissue damage triggered by heat stress in birds [12, 14]. Birds challenge to heat stress have their homeostasis equilibrium impaired between oxidative stress and antioxidant defense mechanisms via increased in the lipid peroxidation and the reduction in enzymatic antioxidants activities [43–45]. The body has a hierarchy of defense tactics to deal with oxidative stress within diverse cellular levels, and overlapped on these are gene-regulated protections encompassing the oxidant stress proteins includes *OVT, AGP, CP, CRP,* and heat-shock proteins [41]. The activities of *GSH-Px, SOD, CAT*, and *TXNRD* antioxidant enzymes were found to increase linearly as clove concentrations increase, indicating an increase in antioxidants level in the present study. The antioxidant equilibrium in biological systems and the scavenging of reactive oxygen species (ROS) are critical for cellular homeostasis [46, 47]. Antioxidant defense is comprised of enzymes and non-enzymatic molecules scavenging responsively on ROS. These includes repair enzymes (GSH-Px, GST, CAT, and DNA repair enzymes) involved in the removal of the oxidative damages. Reactive oxygen species are continuously generated during typical aerobic metabolism that is safely removed by these biological antioxidants. Antioxidant defense is never 100% effective; however, mechanisms of repair are of crucial significance for survival. The results obtained in the current study indicated a significant increase in MDA, GSH-Px, CAT, SOD and TXNRD, and lower concentrations of GSH in the ET-treated fowls as compared to TC. The current findings are consistent with previous results [45, 48]. It is reported that much accumulation of MDA inhibited the antioxidant enzyme activities and accelerated the oxidative damage of DNA and proteins [49]. In the present study, dietary clove supplemented levels 400 and 600 mg in the ET-fowls showed a decrease in MDA levels and an increased in the enzymatic activities (GSH-Px, SOD, CAT, and TXNRD) to a concentrations close to those obtained of TC group. Moreover, the diets supplemented with clove levels improved GSH concentrations, which is an essential mechanism protecting cells from impairment induced by reactive oxygen species (ROS). Furthermore, clove supplementation reduced some of the heat stress-induced effects in the black-meated fowls. The enhancement of antioxidant status of black-meated fowls for clove-supplemented groups suggested that the clove-induced effects were independent of the ET. These results were in agreement with data obtained with other antioxidants of plant source [50].

### Liver and immunological organ growth as it relate to APPs expression

The liver is a vital organ that synthesize APPs and expression of its genes [23, 24]. Similarly, the bursa of Fabricius, thymus, and spleen are significant immunological organs in birds. Out of these, thymus and bursa of Fabricius functions as main lymphoid organs [51, 52], while the spleen serves as primary peripheral immune organ in birds [53]; all these organs are engaged in both humoral and cellular immunity. The expression of mRNA coding for APPs in the liver is commonly controlled at the transcriptional level [35]. The liver regulates the expression of these genes in response to diverse extracellular stimuli [36]. We noted in the current finding that changes in the APPs concentrations were due primarily to the changes in their synthesis by hepatocytes. The amount of this synthesis varies from 35% as regards *CP* to as much as 60% in the case of *AGP* and *SAA*. These changes in plasma concentrations of APPs were preceded by equivalent variations in mRNA levels. Chickens exposed to heat condition have relatively reduced weight of the spleen, bursa of Fabricius, and thymus [54]. The results were in agreement with the result of immunological organs growth in heat-stressed chickens [7]. The present study showed that the growth indices of the liver, spleen, bursa of Fabricius, and thymus in black-meated fowls decreased during 21 d chronic heat stress. However, upon dietary clove supplementation, a significant increase in the indices of the spleen, bursa Fabricius, and thymus were observed in the ET groups. This result suggests that clove as dietary supplements can stimulate the growth of the immune organs and neutralize immune organ dysplasia produced by heat stress.

### Acute phase protein mRNA Expression Levels

Stress increases the synthesis of APPs which are constitutively expressed and play a crucial protective role for DNA against oxidative damage [18, 35, 36] by maintaining the homeostasis and structural integrity of organs over the stress-induced tissue damage [21]. Both low and chronic concentrations of ROS may generate DNA damages by allowing gene mutations and structural modifications into the DNA. Chronic levels of ROS can add to irregular gene expression, obstruct cell-to-cell communications, and alteration of second-messenger structures, thus results in the increase of apoptosis of the initiated cell population.

In this investigation, clove supplementation accelerated the induction of endogenous antioxidant protection system in the black-meated fowls, and also enhanced antioxidant status by mitigates the heat stress-induced APPs expression in the liver and immunological organs. The current results established that the transcription of *OVT, CP, AGP, CRP* and *SAA* were increased in all organs of the black-meated Silkie fowls after 21 d exposure. It was postulated that high rate of APPs translational activity in the liver, spleen, bursa of Fabricius and thymus may have influence the increased of immune organs capacity to provide resistance against oxidative damage. However, when clove was supplemented in the diet, the expression of *OVT, CP, AGP, CRP* and *SAA* genes decreased in the liver, spleen, bursa of Fabricius and thymus. This result was consistent with the APPs mRNA response to ET and the association of these APPs mRNA levels with tissue damage ensued during ET treatment. The present findings are in agreement with the results reported by previous studies [18, 35, 36] in which APR mRNA expression was reduced in the liver tissue. Moreover, the current study revealed that the diet supplemented 400 mg clove maintained *OVT, CP, AGP, CRP* and *SAA* mRNA expression levels in all the organs of heat-stressed Silkie fowls to approximately levels of those fowls maintained at TC. Though, clove induced *OVT, CP, AGP, CRP* and *SAA* mRNA expression in the fowls supplemented this level through activation and mobilization of the organs antioxidant protective mechanisms. Clove as natural polyphenolic bioactive compound has a characteristics of overlapping properties that triggered self-maintenance and defense mechanisms in rats [35, 36, 56]. We deduce that the nature of this mechanism might be a tissue specific at different levels of clove and the patterns of diverse APPs mRNA expression. Conversely, the mechanisms might be controlled by the metabolic and nutrient requirements of these tissues. There are need for the future research to determine the precise and unique mechanisms of actions of the clove supplementation on the immune organs.

### Immunological responses

Clove extract supplementation significantly increase the white blood cell, monocyte and eosinophils concentrations in ET groups. Previously, dietary additive has been found to significantly increase the erythrocyte count, hemoglobin concentration, and hematocrit in turkeys, but the total leucocyte and differential leucocyte counts were not affected by dietary supplementation [62, 63]. White blood cells and other leucocytes are immune-related cells that partake in body defense against external challenge and infections. The current investigation showed that immune-related blood components were improved in fowls supplemented 600 mg clove, however these are not constant with the present data, as observed with other leucocytes. These discrepant data might be ascribed to the type of feed these additive supplemented and the species of the birds used in the studies [63, 64].

Plasma immunoglobulin concentration of the fowls suggests antibodies fight against many immune challenges. The 3 major immunoglobulins are IgG, IgM, and IgA which primarily govern the immune response of an individual. Clove supplemented in the diet of black-meated fowl significantly (*P* < 0.05) elevated the plasma immunoglobulin concentrations (IgG, IgM, and IgA) in the present study. It have been indicated that clove improves immune function and stimulates the synthesis of endogenous antimicrobial peptides in the gut [12, 14, 63]. The enhanced plasma immunoglobulin concentrations of fowls fed 600 mg clove under ET in the current study might be ascribed to the immunomodulatory properties of clove [12, 43, 46, 65]. Though this immunomodulation properties is not fully understood, studies showed that dietary intake of clove can stimulate the diverse parts of the gut-associated immunity along with improved growth performance and antioxidant capacity [12, 43, 50, 65]. Therefore, the ability of clove to enhance the immune system is a viable reason to support their use as an alternative additive for improving bird’s health and productivity under stressful condition. The present study observed that plasma immunoglobulin levels in the fowls significantly increased at 400 and 600 mg clove supplemented groups compared to other groups (0, 200 mg, and control).

## Conclusion

In conclusion, the comparable APPs response and antioxidant enzymes status of both ET and TC Silkie fowls supplemented with and without clove extracts suggested that the mechanisms associated with the positive benefits of this supplementation are not simply associated with the biology of the black-meated fowls but beyond. Clove supplementation improved the growth performance and modulated the immune responses of black-meated Silkie fowls by alleviate the negative effects of heat stress via improvement in antioxidant defense mechanisms. The modulation occurred through enzymatic and non-enzymatic antioxidant systems which have a substantial capacity to scavenged free radicals and induced acute phase proteins in the immune organs and liver. This study has proof that clove extract has a significant antioxidant effect against heat stress in chickens. However, precise mechanisms on how these occur were not attained in the present study. The current results can be adopted as a base for stress study of immune organ growths and APPs mRNA expression to measure the chicken’s immunity during the unpredicted rise in environmental temperatures and the emergence of diseases.

## Additional file

Additional file 1: Bello AU, Sulaiman JA, Aliyu MS. Acute phase protein mRNA expressions and enhancement of antioxidant defense system in Black-meated Silkie Fowls supplemented with clove (Eugenia caryophyllus) extracts under the influence of chronic heat stress, data sets. figshare. 2016.(XLSX 8 kb)

## Abbreviations

AGP: α-1-Acid glycoprotein
APR: Acute phase response
CAT: Catalase
CP: Ceruloplasmin
CRD: Completely randomized design
CRP: C-reactive protein
ET: Elevated temperature
GSH: Glutathione
GSH-Px: GSH peroxidase
MDA: Malonaldehyde
OVT: Ovotransferrin
SAA: Serum amyloid-A
SOD: Superoxide dismutase
TC: Thermoneutral condition
TXNRD: Thioredoxin reductase
